# A safe and potentiated multi-type HPV L2-E7 nanoparticle vaccine with combined prophylactic and therapeutic activity

**DOI:** 10.1038/s41541-024-00914-z

**Published:** 2024-06-26

**Authors:** Xueer Zhao, Yueru Zhang, Oscar Trejo-Cerro, Ecem Kaplan, Zhe Li, Femke Albertsboer, Neyla El Hammiri, Filipe Colaço Mariz, Lawrence Banks, Simone Ottonello, Martin Müller

**Affiliations:** 1 Tumorvirus-specific Vaccination Strategies, German Cancer Research Center, Heidelberg, Germany; 2 International Centre for Genetic Engineering and Biotechnology, Trieste, Italy; 3 B Cell Immunology, German Cancer Research Center, Heidelberg, Germany; 4 Department of Chemistry, Life Sciences and Environmental Sustainability, University of Parma, Parma, Italy

**Keywords:** Protein vaccines, Human papilloma virus

## Abstract

Persistent infection with high-risk human papillomavirus (HPV) is widely recognized as the primary cause of cervical and other malignant cancers. There are six licensed prophylactic vaccines available against HPV, but none of them shows any significant therapeutic effect on pre-existing infections or lesions. Thus, a prophylactic vaccine also endowed with therapeutic activity would afford protection regardless of the vaccine recipients HPV-infection status. Here, we describe the refinement and further potentiation of a dual-purpose HPV nanoparticle vaccine (hereafter referred to as cPANHPVAX) relying on eight different HPV L2 peptide epitopes and on the E7 oncoantigens from HPV16 and 18. cPANHPVAX not only induces anti-HPV16 E7 cytotoxic T-cell responses in C57BL/6 mice, but also anti-HPV18 E7 T-cell responses in transgenic mice with the A2.DR1 haplotype. These cytotoxic responses add up to a potent, broad-coverage humoral (HPV-neutralizing) response. cPANHPVAX safety was further improved by deletion of the pRb-binding domains of E7. Our dual-purpose vaccine holds great potential for clinical translation as an immune-treatment capable of targeting active infections as well as established HPV-related malignancies, thus benefiting both uninfected and infected individuals.

## Introduction

Cervical cancer is the fourth most prevalent cancer among women worldwide. In 2020, there were approximately 604,000 new cases reported, resulting in an estimated 342,000 deaths attributed to cervical cancer^1^. Persistent infection by an oncogenic HPV type is the primary factor leading to the development of cancerous cervical lesions^2^. HPV types 16 and 18, in particular, are estimated to account for approximately 70% of cervical cancer cases^1^. Progression from initial infection to the invasive carcinogenic stage typically takes one to three decades, whereas precancerous lesions tend to develop much earlier^3^.

Even though cervical cancer can be effectively prevented through regular screening and early surgical interventions, the overall burden of the disease, especially in less affluent areas (so-called Low- and Middle-Income Countries, LMIC)^1,4^, has not decreased significantly. Vaccination is regarded as a cost-effective, highly efficient and broadly accessible approach to constrain virus infections and potentially eradicate cervical cancer. At present, there are six licensed prophylactic HPV vaccines available. These include three bivalent vaccines: Cervarix, Cecolin (China only) and Walrinvax (China only); two quadrivalent vaccines: Gardasil and Cervavac (India only); and one nonavalent vaccine: Gardasil 9^1^. All these vaccines are based on the major capsid protein L1 and their mode of action is to induce virus-neutralizing antibodies that typically display a marked HPV-type specificity^5,6^. Until now, no significant therapeutic effect on pre-existing infections and lesions has been documented for these vaccines, despite their excellent prophylactic efficacy in HPV-naïve women^7,8^. Moreover, implementation of national HPV vaccination programs in LMICs, i.e., the very places were preventive screening is less efficient, is subject to several hurdles, such as high vaccine costs, poor stability and complex supply-chains^9^, that severely limit HPV prophylactic programs.

A successful therapeutic vaccination strategy or post-exposure prophylaxis should focus on activating the cellular immune system, e.g. by eliciting virus-specific T-cell responses. The E6 and E7 HPV oncoproteins, which are consistently expressed in both premalignant and invasive lesions^3,10–12^, stand out as prime targets for immunotherapeutic approaches, due to their crucial role in the initiation and advancement of malignancy. Various strategies, including peptide-based, protein-based, viral-vectored, bacterial-vectored, cell-based and DNA/RNA-based vaccine approaches have been employed for the development of HPV therapeutic vaccine prototypes^13^. Some of these have proven to be at least partially successful, but no therapeutic vaccine has yet been licensed for the treatment of premalignant lesions or established HPV associated tumors. In addition, few strategies have been designed for the dual goal of both HPV prevention and immunotherapy of pre-existing HPV-associated lesions^13–19^. Some of these dual-purpose vaccines relied on fusion proteins based on either a multimeric L1-VLP platform^16,17,20,21^, or on an L2 monomeric form comprising the E7/E6 oncoantigens^18,22^. Another vaccine prototype was manufactured and delivered as a DNA formulation. It involves the fusion of HPV16 E6, E7, and L2 with carrier calreticulin (CRT)^19,23^.

In this context, with the aim of conferring therapeutic activity (In the following we use the term ‘therapeutic activity’ for the activation of a cytotoxic T-cell response) to an otherwise prophylactic-only vaccine, we previously incorporated an HPV16 E7-derived MHC-I (H2-D^b^) restricted epitope into an L2 polytope displayed on the surface of a thermostable thioredoxin (Trx), which was then converted into a heptameric nanoparticle format upon fusion with the OVX313 module. The utilized Trx scaffold is derived from the hyper-thermophilic archaeon *Pyrococcus furiosus* showing high resistance to various chemico-physical challenges such as heat, freeze-drying, as well as proteolytic damage, and with a large capacity to accept polypeptide inserts within its display site^24–26^. Based on our testing, we found no detectable humoral immunity against either mouse or human Trx in sera collected from immunized animals or participants involved in the PANHPVAX trial. The multimeric domain, OVX313, is a positively charged derivative originating from the complement system related C4-binding protein (C4bp), which facilitates the independent heptamerization of any protein fusion partner. Moreover, it is a chimeric variant of avian C4-bp sharing less than 20% similarity with human C4-bp, featured to minimize auto-antibody induction^27–33^. Since the L2 polytope-Trx-OVX313-based vaccine^34,35^ (named PANHPVAX) has now entered a phase I clinical study, where it has shown complete safety so far (EudraCT No.: 2021-002584-22), we were prompted to further refine our prophylactic-therapeutic vaccine prototype with the aim of potentiating its cytotoxic T-cell immunogenicity, evaluating the safety of different antigen constructs and testing its activity in humanized transgenic mice expressing the HLA-A2.DR1 haplotype.

The best candidate that emerged from this study, Trx-L2 8mer-HPV16 E7-HPV18 E7-OVX313 (referred to as cPANHPVAX), which incorporates full-length E7 derived from both HPV16 and HPV18, elicits T-cell immunity in the context of the human HLA-A*0201/DRB1*0101 haplotype and while maintaining the humoral HPV neutralizing activity of PANHPVAX, it exhibits a significantly improved therapeutic efficacy compared to our previous antigen design^32^. Furthermore, targeted modification of the E7 inserts, namely deletion of the pRb-binding site, resulted in the complete abrogation of transforming activity even when over-expressed as a genetic vector. Altogether, these data point to the high clinical translation potential of wo/pRb-cPANHPVAX as a vaccine candidate capable of targeting active infections as well as established HPV-related malignancies.

## Results

### Insertion of full-length HPV16 E7 and HPV18 E7 polypeptides to expand the therapeutic spectrum of the dual-purpose vaccine

**Fig. 1:**
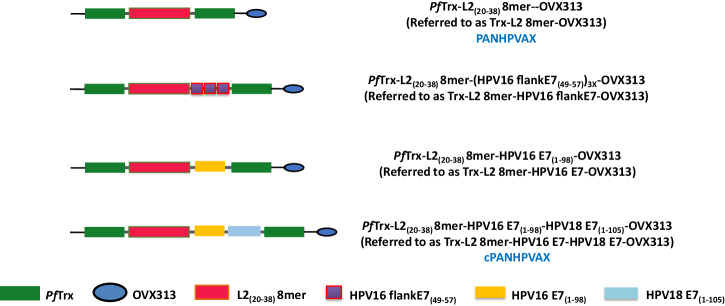
The antigen constructs. PfTrx: Thioredoxin protein derived from the hyper-thermophilic archaeon *Pyrococcus furiosus*^24^. OVX313: recombinant version of the avian C4-binding protein heptamerization domain (OligoDOM technology, OSIVAX^33^). L2 8mer: polytope comprising the L2_(20-38)_ epitopes from eight different HPV types (16-18-31-33-35-6-51-59), selected on the basis of sequence homology to the major cross-neutralization HPV16 L2_(20-38)_ epitope. HPV16 flankE7_(49-57)_: extended E7_(49-57)_ epitope, flanked on both sides by the five amino acids that are located upstream (QAEPD) and downstream (CCKCD) to the sequence of the E7_(49-57)_ epitope (RAHYNIVTF^67^) in E7. Three copies of HPV16 flankE7_(49-57)_ epitopes are present in antigen Trx-L2 8mer-HPV16 flankE7-OVX313. HPV16 E7_(1-98)_: full sequence of HPV16 E7. HPV18 E7_(1-105)_: full sequence of HPV18 E7.

**Fig. 2:**
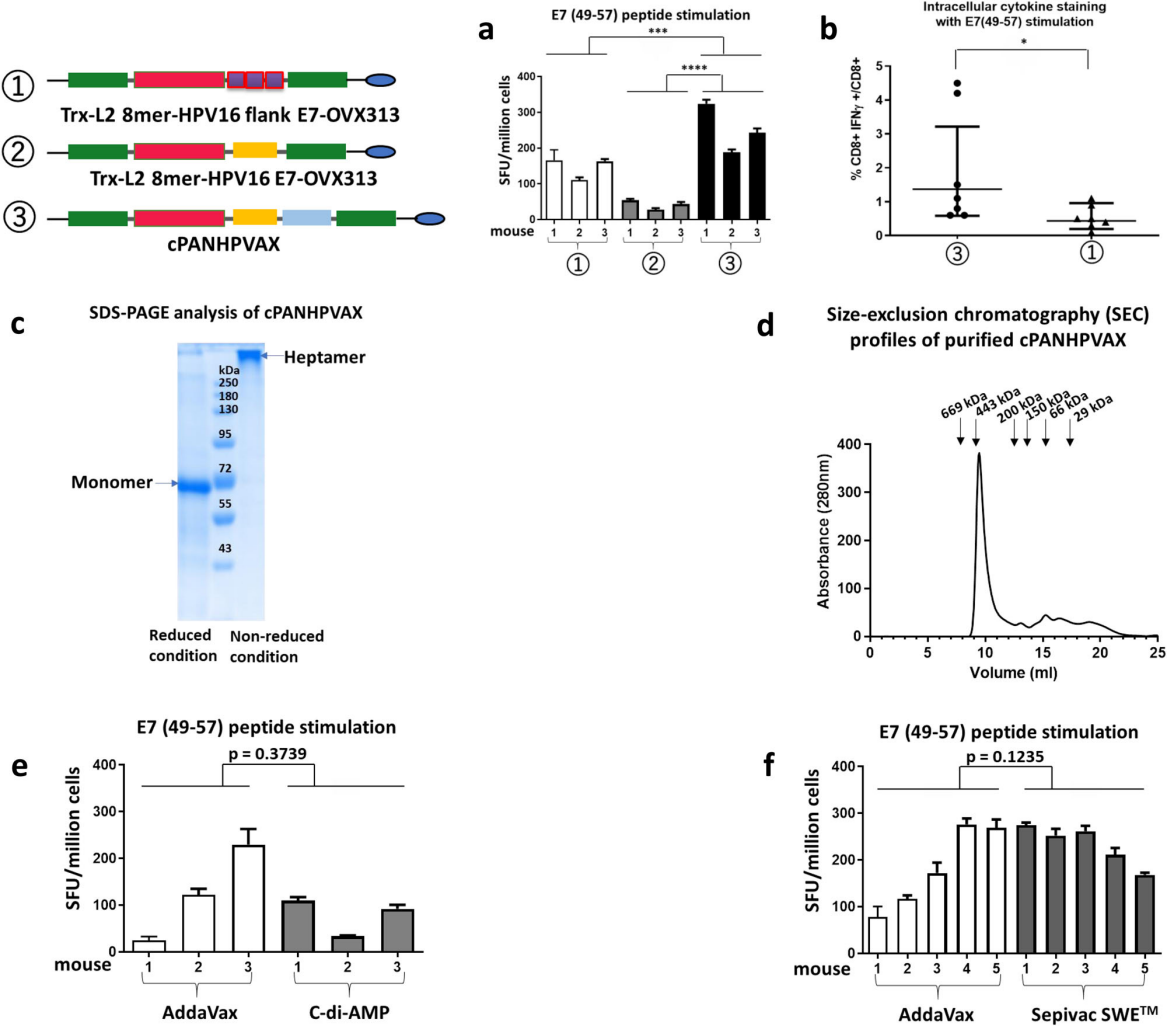
Enhanced anti-HPV16 E7 T-cell responses induced by cPANHPVAX with Sepivac SWE™ as best adjuvant. **a** Numbers of IFN-γ spots per 10^6^ splenocytes (shown as SFU (spot-forming unit)/million cells), measured upon stimulation with the E7_(49-57)_ peptide, were compared among three groups of mice immunized with antigen Trx-L2 8mer-HPV16 flankE7-OVX313 (control), Trx-L2 8mer-HPV16 E7-OVX313 or cPANHPVAX, respectively. The antigens were all adjuvanted with AddaVax. Shown are the mean and SD (standard deviation) of triplicate values for each mouse. Statistical significance (for this assay and the subsequent experiments) was evaluated using the nonparametric Mann–Whitney test. *P*-values ≤ 0.05 are considered as significant and are marked as follows: ***P-value < 0.001; ****P-value < 0.0001. **b** Percentages of IFN-γ-secreting CD8 T-cells are compared between groups of mice immunized with cPANHPVAX or Trx-L2 8mer-HPV16 flankE7-OVX313 (control). Both groups were adjuvanted with AddaVax. Splenocytes were stimulated in vitro with the E7_(49-57)_ peptide. Each dot means a value from one mouse and bars show the mean and SD of 7 mice. *P*-values ≤ 0.05 are considered as significant and are marked as follows: *P-value < 0.05. **c** SDS-PAGE analysis of the purified cPANHPVAX under reducing (left) and non-reducing (right) conditions. The size of the antigen in the monomeric form is 59 kDa and that of the heptamerized structure is 413 kDa. The heptamerization domains are cross-linked via disulfide bridges. **d** SEC profiles of purified cPANHPVAX. Elution profiles of marker proteins are indicated. **e**, **f** Influence of different adjuvant formulations on the antigen cPANHPVAX. Figure 2e: two groups of mice (three per group) were immunized with antigen cPANHPVAX formulated with either AddaVax or C-di-AMP. Figure 2f: five mice per group were immunized with antigen cPANHPVAX formulated with either AddaVax or Sepivac SWE™. The number of IFN-γ spots per 10^6^ spleen cells was measured after stimulation with E7_(49-57)_ peptide. Shown are the mean and SD of triplicate values on each mouse. A *P*-value ≤ 0.05 is considered significant.

We previously developed a dual-purpose vaccine prototype Trx-L2 8mer-HPV16 flankE7-OVX313 harboring, in addition to an HPV-L2 polytope (8mer), three copies of the H2-D^b^-restricted epitope HPV16 E7_(49-57)_, flanked by five amino acids on both sides (flankE7)^32^. The therapeutic aspect of the vaccine therefore was limited to mouse models. With the aim of expanding the therapeutic spectrum of this vaccine, here, we replaced the ‘flankE7’ epitopes with the full-length sequence of the HPV16 E7 polypeptide to generate antigen Trx-L2 8mer-HPV16 E7-OVX313 (Fig. 1). Considering the low sequence similarity (42% identities) between HPV16 E7 and HPV18 E7, which is the second most prevalent oncogenic HPV type, in a second extension, we added the full-length sequence of HPV18 E7, thus generating antigen Trx-L2 8mer-HPV16 E7-HPV18 E7-OVX313 (Fig. 1). Both antigens were expressed in *E. coli*, purified by thermal purification, cation exchange chromatography (CEX), size exclusion chromatography (SEC), and detoxified. In order to select the optimal antigen candidate, three groups of C57BL/6 mice were respectively immunized once with antigen Trx-L2 8mer-HPV16 E7-OVX313, Trx-L2 8mer-HPV16 E7-HPV18 E7-OVX313 or Trx-L2 8mer-HPV16 flankE7-OVX313 (control), formulated with AddaVax (a MF59-like, squalene-based oil-in-water nano-emulsion). Seven days after immunization, splenocytes were analyzed by IFN-γ ELISpot assays using peptide E7_(49-57)_ as stimulant. As shown in Fig. 2a, the Trx-L2 8mer-HPV16 E7-HPV18 E7-OVX313 antigen led to the most robust responses among the three antigens. Also, the responses induced by Trx-L2 8mer-HPV16 E7-OVX313 were apparently lower compared to the control, which, however, harbors three copies of the E7_(49-57)_ epitope. Building upon this result, we conducted a more in-depth analysis of the cytotoxic T-cell immunogenicity of the antigen Trx-L2 8mer-HPV16 E7-HPV18 E7-OVX313 using the intracellular cytokine (IFN-γ) staining (ICS) assay. Two groups of C57BL/6 mice (7 mice/group) were immunized twice at 5 days intervals with antigens Trx-L2 8mer-HPV16 E7-HPV18 E7-OVX313 or Trx-L2 8mer-HPV16 flankE7-OVX313 (control), both formulated with AddaVax. Splenocytes were isolated seven days after the last immunization and the percentage of IFN-γ-secreting CD8 T-cells was determined after stimulation with the E7_(49-57)_ peptide. Also under these assay conditions, antigen Trx-L2 8mer-HPV16 E7-HPV18 E7-OVX313 induced a significantly stronger E7-specific T-cell response compared to the antigen Trx-L2 8mer-HPV16 flankE7-OVX313 (Fig. 2b). Following the confirmation of its OVX313-mediated heptameric structure by SDS-PAGE under reducing and non-reducing conditions as well as by SEC (Fig. 2c, d), we thus chose antigen Trx-L2 8mer-HPV16 E7-HPV18 E7-OVX313 (referred to hereafter as cPANHPVAX) as the leading candidate and investigated its immunogenicity more comprehensively in the subsequent assays.

Next, we tested two different adjuvants, cyclic di-adenosine monophosphate (C-di-AMP; used in the PANHPVAX clinical trial), an agonist of stimulator of interferon genes receptor, STING^36,37^ and Sepivac SWE™ (a human use-approved, GMP-grade analog of AddaVax^38^) in addition to Addavax. The cellular immune responses elicited by cPANHPVAX formulated with each of the three adjuvants were examined in C57BL/6 mice by IFN-γ-ELISpot assays. Seven days after a single immunization, splenocytes were isolated and stimulated in vitro with the E7_(49-57)_ peptide. None of the adjuvants displayed any observable side effects in the animals when combined with cPANHPVAX. Pertaining to the induced immunity, as shown in Fig. 2e, cPANHPVAX formulated with AddaVax induces slightly stronger T-cell responses compared to C-di-AMP. Similarly, the anti-E7 T-cell responses elicited by the Sepivac SWE™ formulation were slightly higher than those induced by the AddaVax formulation (Fig. 2f). Although the differences of immunogenicity among the three adjuvants are not statistically significant, in view of the plan to apply the vaccine in a human genetic background, Sepivac SWE™ was selected as the preferential human compatible adjuvant and adopted in the subsequent experiments.

### Robust and comprehensive humoral immune responses against different high-risk HPV types induced by cPANHPVAX

The prophylactic-only version of our vaccine, PANHPVAX, induces robust humoral immune responses against several mucosal and cutaneous HPV types^35,39^. To examine the impact of E7 insertion on such responses, four doses of cPANHPVAX were injected into Balb/c mice (ten animals/ group), using the standard PANPVAX vaccine as a reference. Following blood collection one month after the last immunization, immune sera from both groups were evaluated by pseudovirion-based neutralization assay (PBNA) and L2-peptide ELISA.

**Fig. 3:**
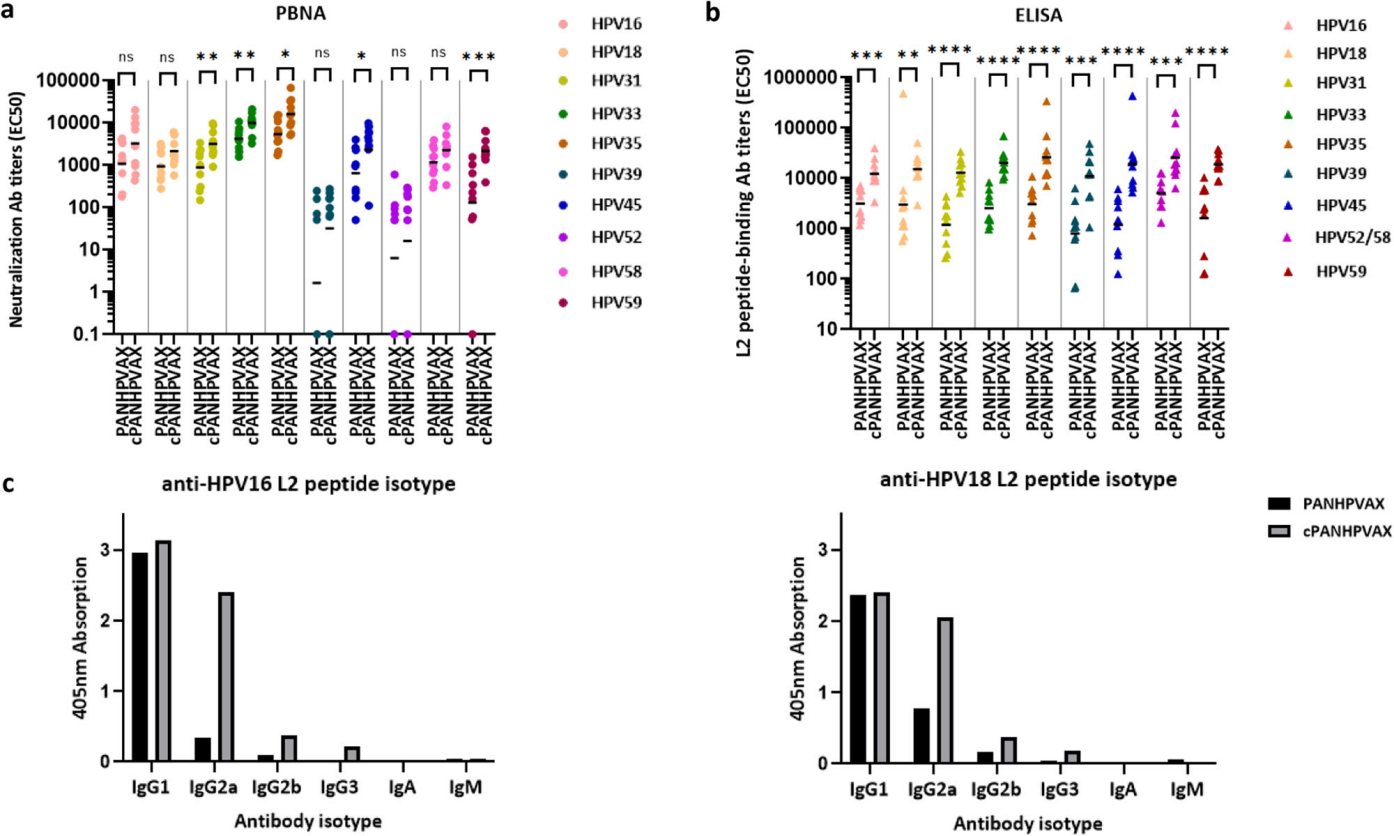
Robust and comprehensive humoral immune response induced by cPANHPVAX. Immune-sera from the indicated groups (10 animals/group), collected one month after the last immunization with either cPANHPVAX or the prophylactic-only PANHPVAX vaccine, were analyzed by PBNA and L2-peptide ELISA. **a** Neutralizing antibody levels against HPV16, HPV18, HPV31, HPV33, HPV35, HPV39, HPV45, HPV 52, HPV58 and HPV59 PsV measured by PBNA. Each symbol represents the neutralizing antibody titer (EC50) determined in an individual mouse serum; geometric means of the titers for each group are indicated by the horizontal lines. **b** L2-peptide ELISA. Each symbol represents the L2 peptide-binding antibody level (EC50) measured in an individual mouse serum; the horizontal lines indicate geometric means of the titers for each group. Statistical significance, determined by the Mann–Whitney test, is indicated as follows: *p-value ≤ 0.05; **p-value ≤ 0.01; ***p-value ≤ 0.001; ****p-value ≤ 0.0001. ns non-significant. Neutralizing antibody titers lower than 50 were considered as non-neutralizing and set at baseline, 0.1 level. **c** An analysis of the isotype distribution of L2 peptide-binding antibodies was conducted using antibody isotype ELISA, with values obtained from the pooled sera of the different mice sera groups.

Neutralizing antibody titers against ten HPV pseudovirion (PsV) types were determined for both antigen groups using PBNA (Fig. 3a). Overall, neutralizing antibody levels elicited by cPANHPVAX exceeded those induced by the standard PANHPVAX vaccine, with statistical significance for some HPV types. In case of HPV31, for example, neutralizing antibody levels induced by cPANHPVAX were significantly higher than those induced by PANHPVAX (*p*-value = 0.0068). Similarly, anti-HPV33 and anti-HPV35 neutralizing antibody levels in the cPANHPVAX group were on average three-fold higher than in the PANHPVAX group (*p*-value < 0.05). The cPANHPVAX also featured significantly higher anti-HPV45 (*p*-value = 0.0355) and anti-HPV59 (*p*-value = 0.0001) neutralizing antibody levels compared to the PANHPVAX group.

As shown in Fig. 3b, antibody titers were also quantified by ELISA, using a set of nine different L2 peptides. This set of peptides actually represents ten HPV types as the HPV52 and HPV58 L2_(20-38)_ peptide sequences are identical. In general, all immune sera were found to be reactive with all the nine L2 peptides. Although intragroup variability of antibody titers was higher than that observed with the PBNA, all sera from the cPANHPVAX group displayed significantly more elevated L2 ELISA titers compared to sera from the reference PANHPVAX group. More specifically, geometric mean values for each of the cPANHPVAX-immunized groups were 4- to 12- fold higher than the corresponding values measured in the PANHPVAX groups (Fig. 3b).

Statistically significant positive Spearman correlations between PBNA and L2-ELISA data were observed for all the different HPV-types, except HPV52 (see Supplementary Fig. 1). In particular, a fairly strong positive correlation coefficient (r) > 0.75, was determined in assays for HPV31, HPV39, HPV45 and HPV59, whereas a moderately positive correlation coefficient, with *r*-value ranging from 0.5 to 0.75, was obtained for HPV16, HPV18, HPV33, HPV35 and HPV58.

The isotype profile of anti-HPV16 and anti-HPV18 antibodies induced by the two antigens (cPANHPVAX and PANHPVAX) was also determined by ELISA (Fig. 3c). Both antigens predominantly elicited IgG1 and IgG2 isotypes, and a more robust induction of IgG2a antibodies was observed in the cPANHPVAX group.

### cPANHPVAX-induced T-cell responses in humanized HLA-A2.DR1 transgenic mice

**Fig. 4:**
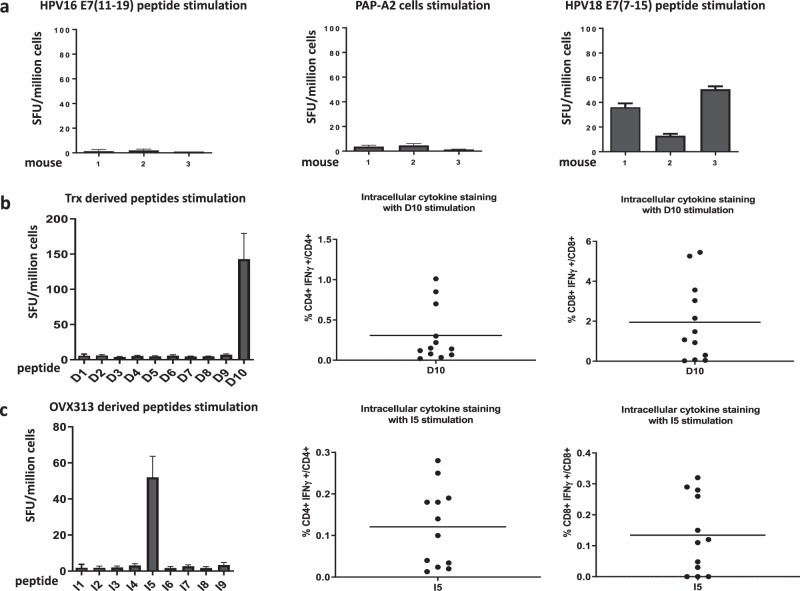
cPANHPVAX-induced T-cell responses in humanized A2.DR1 transgenic mice. A2.DR1 mice were immunized twice at 5 days intervals with antigen cPANHPVAX formulated with Sepivac SWE™. Splenocytes were harvested seven days after the second immunization and stimulated ex vivo with the corresponding peptides or syngeneic tumor cell line. IFN-γ-producing T cells were analyzed using ELISpot or ICS assay. **a** cPANHPVAX can induce HPV18 E7-specific cytotoxic T-cell responses in A2.DR1 mice. Numbers of IFN-γ spots per 10^6^ splenocytes were determined in three immunized A2.DR1 mice. Splenocytes from pre-immunized mice were stimulated with the HPV16 E7_(11-19)_ peptide, PAP-A2 cells or the HPV18 E7_(7-15)_ peptide. Shown are the mean and SD of triplicate values determined on each mouse. **b** Trx scaffold-derived T-cell responses can be detected in A2.DR1 mice. Numbers of IFN-γ spots per 10^6^ splenocytes (left panel) were determined by ELISpot assay upon stimulation with the D1 to D10 peptides. Shown are the mean and SD of triplicate values obtained with four mice. In the ICS assay, the percentage of IFN-γ-secreting CD4 T-cells and the percentage of IFN-γ-secreting CD8 T-cells were determined after stimulation with the D10 peptide. Individual dots represent the values measured for each mouse; horizontal bars indicate the geometric means derived from twelve mice. The example of gating strategies in flow cytometry is illustrated in Supplementary Fig. 3. **c** OVX313 domain-derived T-cell responses can be detected in A2.DR1 mice. Numbers of IFN-γ spots per 10^6^ splenocytes (left panel) were determined by ELISpot assay upon stimulation with the I1 to I9 peptides. Shown are the mean and SD of triplicate values obtained with six mice. In the ICS assay, the percentage of IFN-γ-secreting CD4 T-cells and the percentage of IFN-γ-secreting CD8 T-cells were determined after stimulation with the I5 peptide. Individual dots represent the values measured in each mouse; horizontal bars indicate the geometric means derived from twelve mice.

To assess cPANHPVAX efficacy in the context of a human MHC haplotype, we investigated the cytotoxic and T-helper responses induced by this vaccine candidate in an A2.DR1 transgenic mouse model^40–42^. The anti-E7 T-cell responses were first analyzed by IFN-γ ELISpot assays, using splenocytes collected seven days after the second immunization and stimulated ex vivo with the HPV16 E7_(11-19)_ peptide (YMLDLQPET, containing an HLA-A2.1-restricted epitope)^43^, the HPV16 E7-expressing syngeneic tumor cell line PAP-A2^44^ or HPV18 E7_(7-15)_ peptide (TLQDIVLHL, containing an HLA-A2.1-restricted epitope)^45^, respectively. As shown in Fig. 4a, only weak T-cell responses were detected with splenocytes stimulated with the HPV16 E7_(11-19)_ peptide or the E7-positive PAP-A2 cell line. A significantly more sustained T-cell response was observed upon stimulation with the HPV18 E7_(7-15)_ peptide.

Next, we set out to examine CD4 + T-helper responses elicited by immunization with the cPANHPVAX vaccine. The ‘SYFPEITHI‘ online software was used to predict HLA-DR1-restricted epitopes (15 amino acids-long) in the cPANHPVAX polypeptide sequence. We selected 10 peptides (named D1 to D10) whose scores were equal to, or higher than 30 (Supplementary Table 1). Following two immunizations of A2.DR1 mice with cPANHPVAX at 5 days intervals, splenocytes were isolated and stimulated ex vivo with the D1-D10 peptides panel and IFN-γ-producing T cells were determined with ELISpot assays. As shown in Fig. 4b (left panel), only the D10 peptide, which is located within the Trx scaffold, was able to trigger a T-cell response. Likewise, and in accordance with our previous findings^32,35^, we found that another peptide, designated as I5 (one of nine peptides located within the OVX313 oligomerization domain; see Supplementary Table 2), stimulated a T-cell response, as revealed by IFN-γ ELISpot assays performed on splenocytes isolated from cPANHPVAX-vaccinated A2.DR1 mice (Fig. 4c, left). Given the large size of the stimulating D10 (15 amino acids) and I5 (20 amino acids) peptides, we asked whether they contained both CD4 and CD8 epitopes. We addressed this question by performing IFN-γ ICS assays, which, as shown in Fig. 4b, c, revealed the induction of both CD4 and CD8 T-cell responses upon stimulation with the D10 and I5 peptides. The D10 peptide proved to be a significantly more effective stimulant than peptide I5 and, quite surprisingly, its CD8 response was approximately 7-fold higher than the corresponding CD4 response. Although effective HPV (L2 and E7) T-cell epitopes in addition to HPV18 E7_(7-15)_ remain to be identified, the above data indicate that the Trx and OVX313 scaffolds comprise human HLA-DR1-restricted CD4 T-helper and HLA-A2.1-restricted CD8 T-cytotoxic epitopes.

### An enhanced-safety variant of cPANHPVAX with a deleted E7 pRb-binding site

**Fig. 5:**
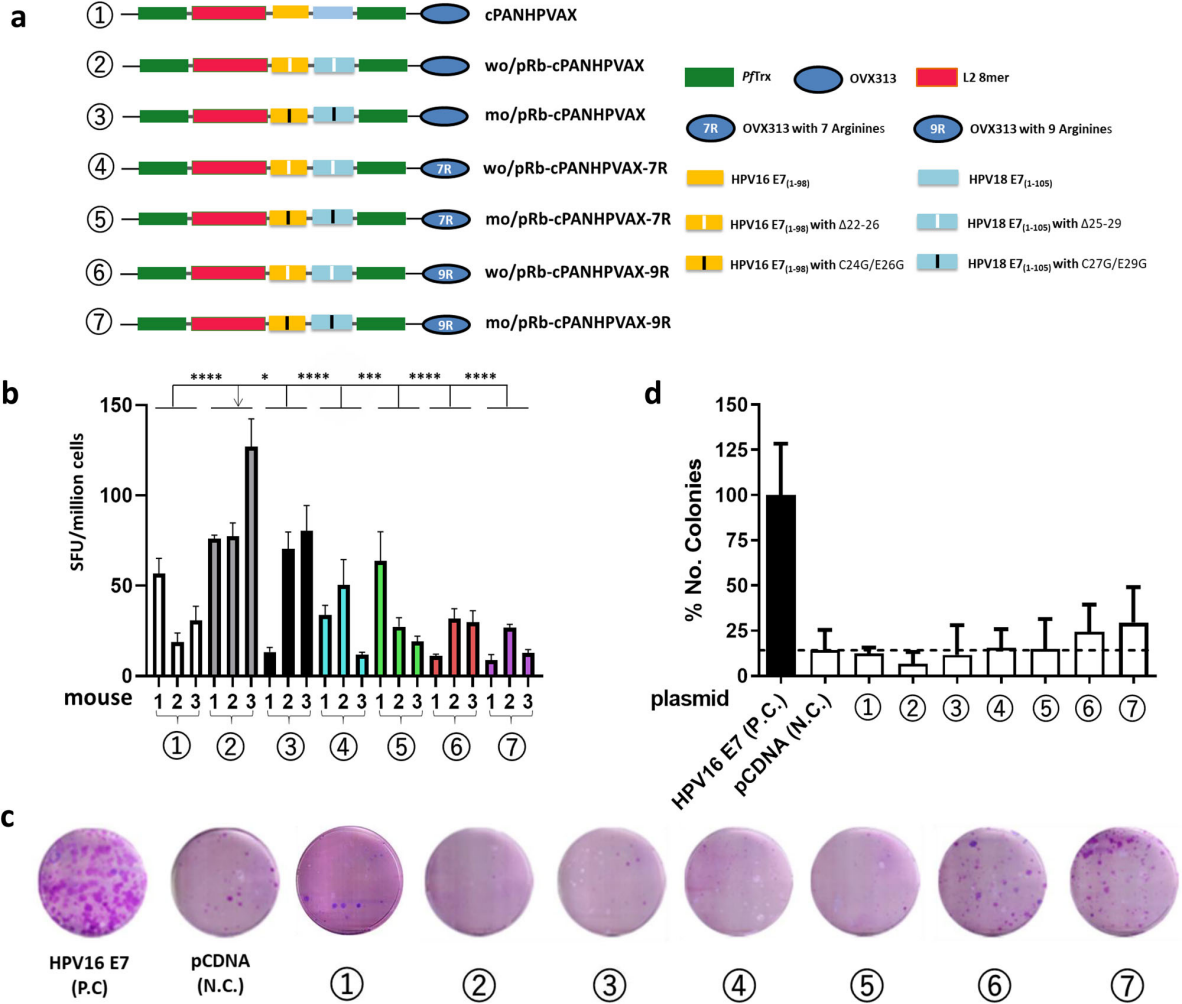
Increased safety and translational potential of a cPANHPVAX variant lacking the E7 pRb-binding sites. **a** Schematic representation of the cPANHPVAX antigen variants **b** Numbers of IFN-γ spots per 10^6^ splenocytes, measured upon stimulation with the E7_(49-57)_ peptide, were compared in 7 groups of mice immunized with antigen cPANHPVAX, wo/pRb-cPANHPVAX, mo/pRb-cPANHPVAX, wo/pRb-cPANHPVAX-7R, mo/pRb-cPANHPVAX-7R, wo/pRb-cPANHPVAX-9R or mo/pRb-cPANHPVAX-9R, respectively. Shown are the mean and SD of triplicate values on each mouse. Statistical significance was assessed using the nonparametric Mann–Whitney test. *P*-values ≤ 0.05 are considered as significant and are labeled as follows: *P-value < 0.05; **P-value < 0.01; ***P-value < 0.001; ****P-value < 0.0001. **c** The BRK assay was performed to evaluate the oncogenic transforming potential of different antigen candidates. All the genes encoding antigen candidates were cloned into pcDNA 3.1 (+) under the control of the cytomegalovirus (CMV) immediate early promoter. The BRK assay was performed by using the 7 different pcDNA 3.1 (+) based constructs (3 independent experiments), co-transfected with EJ-ras. The plasmid encoding HPV16 E7 is used as a positive control (P.C.). The empty vector pcDNA 3.1 (+) is used as a negative control (N.C.). **d** Relative quantification of the results of the BRK assay. The number of colonies is expressed as the percentage of colonies obtained in cells transfected with plasmids HPV16 E7 and EJ-ras, which is taken as 100%.

Binding of E7 oncoproteins to the retinoblastoma tumor suppressor protein (pRb) correlates with the transforming capacity and carcinogenic potential of high-risk HPVs^46–48^. Even though cPANHPVAX is a post-translationally unmodified recombinant fusion protein and produced in *E.coli* under control of the bacteriophage T7 promoter, we wanted to add an additional safety level by targeting the pRb binding domains in HPV16 E7 and HPV18 E7. As illustrated in Fig. 5a, the pRb binding sites were either deleted (Δ22-26 and Δ25-29 amino acids in the case of HPV16 and HPV18 E7, respectively) to generate antigen wo/pRb-cPANHPVAX, or sequence-modified (C24G/E26G and C27G/E29G in the case of HPV16^49^ and HPV18^50^ E7, respectively) to generate antigen mo/pRb-cPANHPVAX.

In addition to these modifications, we investigated whether an increase of the net positive charge of the antigen might improve its immunogenicity or ease/yield of production. In fact, positive supercharging, by promoting cellular penetration, has previously been reported to improve immunogenicity and other features of various antigens^51–56^. Both E7 proteins contain a significant number of acidic amino acids while the OVX313 heptamerization domain contains a cluster of five arginine (R) residues presenting positive charges on the antigen^33^. In order to expand the net positive charge of the antigen, we extended the arginine cluster of OVX313 by two or four R residues, thus generating four antigen variants (Fig. 5a): wo/pRb-cPANHPVAX-7R, mo/pRb-cPANHPVAX-7R (two ‘R’ extension) and wo/pRb-cPANHPVAX-9R, mo/pRb-cPANHPVAX-9R (four ‘R’ extension). All the antigen candidates were purified through thermal treatment, CEX and SEC (see SDS-PAGE profiles in Supplementary Fig. 2). The induced T-cell immunity among the produced antigen variants was assessed by IFN-γ ELISpot assay in C57BL/6 mice. We observed that the anti-E7 T-cell response induced by antigen wo/pRb-cPANHPVAX is significantly higher than that induced by other antigen candidates (Fig. 5b).

To determine the impact of the Rb mutations, we evaluated the oncogenic potential of cPANHPVAX and its variants by a cell-based transforming assay in baby rat kidney cells (BRK)^57^. All cPANHPVAX variants were expressed from the CMV immediate early promoter. Cells were co-transfected with the different antigen constructs and EJ-ras. A representative colony image is shown in Fig. 5c. The number of colonies is expressed as the percentage of colonies obtained in cells transfected with expression plasmids for HPV16 E7 (positive control) and EJ-ras, which is taken as 100% (Fig. 5d). We can see in the histogram that, even when expressed under the control of CMV promoter, the antigen constructs are not transforming BRK cells, or they present a drastically reduced ability to induce cell transformation compared to the positive control. Considering together with the robust induced T-cell responses, the antigen wo/pRb-cPANHPVAX (#2 in Fig. 5a) stands out as our lead vaccine candidate.

## Discussion

HPV prophylactic vaccines have been available for several years, yet vaccine coverage worldwide, which is estimated to be around 20%, remains far below the aimed coverage of 90%^58^. This disparity is particularly striking in less affluent countries, where the implementation of nation-wide vaccination programs has been constrained by several factors. Consequently, in 2020, approximately 90% of new cases and HPV-related deaths globally were concentrated in LMICs^59^. Furthermore, a substantial number of HPV-infected women is expected to develop precancerous and cancerous cervical lesions in the near future. This grim scenario attests to the urgent need for a dual-purpose, preventive and therapeutic, HPV vaccine. Such a vaccine, because of its ability to target various HPV-related lesions, holds substantial value, especially in the context of post-exposure prophylaxis.

The key to eliminating virus-infected cells lies in anti-early HPV-encoded proteins, particularly anti-E6 or anti-E7 T-cell responses. There is no clear evidence to date supporting the notion that humoral, B-cell-mediated antibody responses directed against capsid proteins have any impact on countering the HPV-infected state. As virus-infected cells progress through the epithelial differentiation program, a shift in viral gene expression occurs, leading to over-expression of virus capsid proteins and subsequent virion formation in the upper layers of the epithelium, accompanied by skin shedding and virus spreading. Since cellular immunity does not play a prominent role during this specific phase of virus replication, infection of nearby epithelial cells by these newly released virions can only be prevented through humoral antibody responses directed against capsid proteins. Therefore, we believe that simultaneously stimulating B- and T-cell responses is of paramount importance in order to establish an effective immunity against the intricate and dynamic chain of events that characterize the HPV life cycle.

Moreover, following virus clearance, dual-purpose vaccines might be able to protect epithelial cells from recurrent HPV infections. Recognizing the broad scope and potential advantages of such a comprehensive anti-HPV approach has spurred the exploration of vaccines that can elicit both prophylactic and therapeutic effects against HPV.

Several HPV antigens have been explored in this context, with a handful of them currently undergoing clinical trial investigations. One notable candidate, TA-CIN (Tissue Antigen-Cervical Intraepithelial Neoplasia), is a fusion protein made up by tandemly linked full-length of HPV16 E6, E7, and L2 antigens. Preclinical studies have demonstrated that TA-CIN triggers a T-cell immune response against HPV16 E6/E7, while stimulating the production of L2-specific neutralizing antibodies^18^. Ongoing clinical trials (NCT02405221) are evaluating the possible use of TA-CIN in patients with HPV16-associated cervical cancer. Another promising candidate is a DNA vaccine named hCRTE6E7L2, which consists of HPV16 E6, E7, and L2_(11-200)_ polypeptides fused to calreticulin (CRT). Encouraging preclinical data have shown that immunization with hCRTE6E7L2 DNA can induce the production of HPV16 E6- and E7-specific CD8 + T-cells, along with protective anti-HPV16 L2 antibodies^19^. Also, this vaccine prototype is currently undergoing clinical investigation (NCT03913117 and NCT04131413).

In our study, we developed a dual-purpose HPV vaccine, designated as cPANHPVAX, based on multi-type L2 and E7 antigenic modules. To broaden the therapeutic scope of cPANHPVAX and facilitate its application in research models with different genetic/immunological backgrounds, we decided to incorporate the full-length version of the HPV16 and HPV18 E7 oncoproteins.

In C57BL/6 mice, cPANHPVAX elicited a significantly enhanced E7_(49-57)_-specific T-cell response compared to our previous, ‘control’ antigen (Trx-L2 8mer-HPV16 flankE7-OVX313; Fig. 2a, b). Notably, while the control antigen contains three copies of the E7_(49-57)_ epitope, cPANHPVAX only carries a single copy of this epitope. The enhanced T-cell immunogenicity observed with cPANHPVAX may thus be attributed to putative multiple helper T-cell epitopes present in the full-length version of the E7 proteins. This view is consistent with the high IgG2 titers induced by the vaccine (Fig. 3c), which are associated with the sustained Th1 responses that are crucial for cell-mediated immunity. In our previous work, we demonstrated that the HPV16 flankE7 control antigen promotes tumor regression in a mouse model of HPV16-induced carcinogenesis^32^. Even though this kind of in vivo testing was not performed in the present study, the superior CTL immunogenicity of cPANHPVAX (Fig. 2a, b) strongly suggests a similarly potent therapeutic effect in a HPV-induced tumor mouse model.

Proteins undergo processing by antigen-presenting cells, leading to the display of different epitopes via MHC class I and class II molecules, generating CD8+ cytotoxic and CD4+ helper T-cell responses without haplotype restriction. Indeed, cPANHPVAX administration resulted in an anti-HPV18 E7 T-cell response in humanized, HLA-A2.DR1 expressing transgenic mice, upon stimulation with the HPV18 E7_(7-15)_ peptide (Fig. 4a). Unfortunately, no T-cell response was observed following splenocyte stimulation with the HPV16 E7_(11-19)_ peptide or the E7-positive PAP-A2 cell line. This negative result may be explained by the lack of adequate processing and MHC-I-mediated presentation of the HPV16 E7_(11-19)_ peptide. The identification of HLA-restricted HPV16 E7 T-cell epitopes may necessitate a more comprehensive epitope mapping and subsequent testing.

The IFN-γ ELISPOT-positive peptide D10, located within the Trx scaffold, was predicted to be both HLA-DR1- and HLA-DR4-restricted. While T-cell immunogenicity of this peptide was evaluated and confirmed in the HLA-DR1, human-like context of transgenic A2.DR1 mice, a similar evaluation in a HLA-DR4 genetic background will have to await the availability of a HLA-DR4 transgenic mouse model.

Two distinct assays, PBNA and L2-peptide ELISA, were used to assess the humoral immunogenicity of cPANHPVAX. Of note, far from interfering with humoral immunogenicity, the incorporation in the cPANHPVAX antigen of full-length HPV16 E7 and HPV18 E7 polypeptides resulted in higher titers of L2-specific and PsV neutralizing antibodies compared to the standard PANHPVAX vaccine (Fig. 3a, b). We hypothesize that this increased humoral immunogenicity is driven by the enhanced T-helper responses arising from the E7 polypeptides. This is reflected in the elevated IgG1 and IgG2 levels measured in immune-sera from cPANHPVAX- compared to PANHPVAX-vaccinated animals (Fig. 3c). Particularly noticeable were the apparently higher IgG2a titers found in cPANHPVAX-vaccinated mice, which are indicative of a potent Th1 response associated with enhanced humoral and cellular immunogenicity.

Positive Spearman correlations were observed between neutralizing and L2 peptide-binding antibody titers across all HPV types, except for HPV52 (Supplementary Fig. 1). A stronger correlation points to higher vaccine effectiveness, thus indicating that most vaccine-induced anti-L2 antibodies are also virus-neutralizing antibodies. In this context, the less labor-intensive L2-peptide ELISA lends itself as a valuable alternative to the more sophisticated PBNA, at least for a preliminary L2-oriented evaluation of antibody-mediated HPV neutralization. The poor correlation we observed with HPV52, may suggest that antibodies raised against this particular HPV type are predominantly non-neutralizing, or, alternatively, that the HPV52 pseudovirions utilized for the PBNA do not allow to detect L2-induced neutralizing antibodies. In line with this assumption, we have recently demonstrated that the L2-peptide ELISA better correlates with neutralization assays using furin-cleaved PsV particles compared to standard PsV, and this is particularly relevant for HPV52^60^.

The anti-E7 T-cell responses observed in both C57BL/6 and humanized, HLA-A2.DR1 transgenic mice, along with the concomitant induction of HPV neutralizing antibodies, point to cPANHPVAX as a promising vaccine candidate that can be beneficial to uninfected as well as early infected individuals. In addition, vaccine efficacy is further reinforced by the two scaffold polypeptides, Trx and OVX313, which effectively stimulate CD4 and CD8 T-cell responses in A2.DR1 mice (Fig. 4b, c).

Our dual-purpose vaccine, with the ultimative goal to alleviate the social burden of HPV infection on global health, holds distinct advantages over similar vaccines. Manufactured in *E. coli*, a widely used and cost-effective host for recombinant antigen production, cPANHPVAX utilizes Trx from the hyper-thermophilic archaeon *Pyrococcus furiosus*. Thanks to this robust scaffold, our vaccine does not require a cold-chain storage/distribution system, which results in significant cost savings and makes it easily accessible to lower income countries. Regarding prophylaxis, specifically, the L2 polytope present in cPANHPVAX stimulates the production of cross-protective antibodies capable of neutralizing not only all high-risk HPV types but also several cutaneous types. In the realm of therapeutics, two distinct E7 polypeptides utilized in the vaccine are derived from the most clinically prevalent HPV types, with the aim of broadening its therapeutic effectiveness.

In view of clinical translation, we measured vaccine-associated transforming activity and introduced targeted sequence modifications into the E7 polypeptides, in order to verify and improve the safety of cPANHPVAX. Previous studies have suggested that mutations at pRb binding site drastically reduced the ability of E7 to induce cell transformation^61,62^. Consistent with this, our findings show that cPANHPVAX and its variants with mutations at the pRb site are not able to transform cells and maintain its CTL immunogenicity (Fig. 5).

Positive supercharging of the antigen with the use of cell-penetrating peptides/proteins or by polycationic resurfacing of the scaffold has previously been shown to enhance immunogenicity of various vaccines^63,64^. This strategy has been successfully applied also to an HPV-L2 polytope displayed on the surface of an engineered, positively supercharged variant ( + 21) of PfTrx^56^ as well as to an HPV16 E7 DNA vaccine^51^. Considering the overall negative charge associated to the E7 oncoproteins and the presence of a previously engineered arginine (R) cluster in the C-terminal region of OVX313, we decided to further expand this cluster (Fig. 5a) and test the effect of positive supercharging on cPANHPVAX immunogenicity. Contrary to our expectations, expansion of the OVX313 arginine cluster by two or four R residues failed to improve, and actually lowered, cPANHPVAX CTL immunogenicity (Fig. 5b), with only a marginally positive effect on the yield of recombinant antigen purification by CEX chromatography (not shown). As suggested by the reduced amounts of the fully assembled 7 R antigen variant revealed by non-reducing SDS-PAGE (Supplementary Fig. 2), this negative result is likely due to interference of the expanded arginine cluster on OVX313-mediated heptamerization. Similar results were obtained with the further supercharged 9 R variant of OVX313 (data not shown).

Based on the above data (summarized in Fig. 5), we selected the standard (OVX313-5R-containing) version of the pRb binding site-deleted cPANHPVAX antigen (wo/pRb-cPANHPVAX) as the best dual-purpose vaccine candidate that emerged from our study. Regarding the vaccine-induced B-cell immunity, we anticipate that a modification in the E7 part, particularly with a minor change from cPANHPVAX to wo/pRb-cPANHPVAX, will not result in a diminished humoral response. This perspective is substantiated by comparing Trx-L2 8mer-HPV16 flankE7-OVX313 with PANHPVAX (referred to as Trx-8mer-OVX313 in the previous publication)^32^, as well as cPANHPVAX with PANHPVAX (as shown in Fig. 3a, b).

Incorporation in our dual-purpose vaccine candidate of nearly full-length E7 polypeptides from two different HPV types was aimed at expanding its therapeutic action spectrum. The two chosen HPVs (types 16 and 18) are the most clinically represented among the over 15 oncogenic HPV types. Insertion of E7 proteins from additional HPV types will likely affect production yield and solubility. It is not known if different E7 proteins share CD8 T-cell epitopes, therefore a comprehensive therapeutic efficiency against all oncogenic HPV cannot readily be expected. On the other hand, we believe that the remarkably broad prophylactic capacity afforded by the L2 polytope can compensate for a certain degree for the limited set of HPV E7 polypeptides present in our vaccine. Based on the promising results reported in this study, we are currently working on clinical translation towards a GMP-grade form of wo/pRb-cPANHPVAX as further improved purity is required for the application in clinical studies. The fact that Sepivac SWE™, which is available in a human-use-approved GMP-grade form, emerged as the best adjuvant in our assays, will certainly ease this transition.

## Methods

### Ethics statement

C57BL/6 N mice, A2.DR1 mice and BALB/c mice at the DKFZ are kept in compliance with German and European statutes and all animal experiments were carried out with the approval of the responsible Animal Ethics Committee (Regional Council of Karlsruhe, Germany; 35-9185.81/G-20/17, 35-9185.81/G-20/22 and 35-9185.81/G-248/16).

### Protein expression and purification

PANHPVAX was provided by Biomeva GmbH (Heidelberg, Germany). The detailed purification procedure was described previously^60^. Other antigen candidates were produced in the lab. Briefly, synthetic DNA encoding target proteins were inserted into the pET 24 plasmid for expression in *E. coli* BL21. The antigen proteins were purified by three serial procedures. First, the antigens were isolated by thermal purification of a temperature range from 60 °C to 70 °C. Then the cation exchange chromatography (HiTrap SP FF column, GE Healthcare) based on an arginine-rich motif at the C-terminus of the OVX313 heptamerization domain (OligoDOM technology, OSIVAX) was performed. The size exclusion chromatography was applied as a refinement step. The concentration and purity of the proteins were analyzed by SDS-PAGE–Coomassie blue staining. All protein samples presented in the same SDS-PAGE were processed in parallel and derived from the same experiment. For endotoxin removal, all proteins were detoxified twice by Triton X-114 separation before immunization. We have verified the effective elimination of endotoxin using this approach by assessing the protein preparations employed in the study. According to the Limulus amebocyte lysate test, the resulting endotoxin levels (0.45-0.8 EU endotoxin/dose) are below the permissible limits for preclinical research^65^ and are also comparable among antigen samples, indicating that variations in observed immunogenicities are not attributed to differences in endotoxin levels.

### Mouse immunization

The 6 to 8 weeks-old female C57BL/6 N mice were purchased from Envigo (Gannet, France; animal permit G20/17 or G20/22, Regierungspräsidium Karlsruhe, Germany) and the 6 to 8 weeks-old female BALB/c mice were obtained from Charles River Laboratories (Sulzfeld, Germany; animal permit G248/16, Regierungspräsidium Karlsruhe, Germany). Mice were kept at the German Cancer Research Center under specific-pathogen-free conditions. The A2.DR1 mouse model^42,66^ (transgenic for HLA-A*0201 and HLA-DRB1*0101) was provided by the Institut Pasteur (Paris, France) and bred in the German Cancer Research Center under specific-pathogen-free conditions. The mice used in the assays were males, 8 to 10 weeks old. C57BL/6 N and A2.DR1 mice were used for assessment of cellular immune responses. For C57BL/6 N mice, 20 μg of the protein adjuvanted with 50% (vol/vol) AddaVax (InvivoGen), 50% (vol/vol) Sepivac SWE™ (Seppic and Vaccine Formulation Institute^38^) or 7.5 μg C-di-AMP (ASA Spezialenzyme GmbH) in a total volume of 100 µl was injected subcutaneously into the base of a mouse tail. A single injection was administered and mice were sacrificed in a CO_2_ chamber 7 days later for ex vivo analysis of T-cell responses by IFN-γ ELISpot assay. Alternatively, C57BL/6 N mice were immunized twice at 5 days intervals and splenocytes of sacrificed mice were analyzed by IFN-γ ICS assay 7 days after the last immunization. Regarding to the A2.DR1 mice, 20 μg of the protein adjuvanted with 50% (vol/vol) Sepivac SWE™ were applied twice at 5-day intervals in a volume of 100 µl into the base of a mouse tail subcutaneously. IFN-γ ELISpot and IFN-γ ICS assays were performed at 7 days after the last immunization to analyze the induced T-cell responses. Female BALB/c mice were employed for the evaluation of humoral immune responses. Twenty μg of protein adjuvanted with 50% (vol/vol) AddaVax in a volume of 50 µl were injected intramuscularly into the caudal thigh muscle. Mice were immunized 4 times at biweekly intervals. One month after the last immunization, the animals were sacrificed using a CO_2_ chamber. Blood was collected by cardiac puncture and serum was analyzed by PBNA, L2-peptide ELISA and antibody isotype ELISA.

### Enzyme-linked immunosorbent spot assay (ELISpot)

Splenocytes isolated from vaccinated mice were plated at 1 million cells per well with 100 ng/ml of the synthetic peptide HPV16 E7_(49-57)_ (GenScript), or 1 μg/ml of the synthetic peptides HPV16 E7_(11-19)_ or HPV18 E7_(7-15)_ (GenScript), or 5 μg/ml of ‘SYFPEITHI‘-predicted HLA-DR1-restricted-epitope peptides D1 to D10 (referred to Supplementary Table 1) (GenScript), or 10 μg/ml of OVX313 overlapping peptides I1 to I9 (referred to Supplementary Table 2) (Mimotopes), or HPV16 E7-expressing syngeneic tumor cell line PAP-A2 in anti-mouse IFN-γ (1:200 dilution in PBS, # 551216, BD Pharmingen)-coated Multiscreen IP plates (Merck Millipore). Concanavaline A (10 μg /ml) and un-stimulated splenocytes were used as positive and negative controls, respectively. Secreted IFN-γ was detected using biotinylated anti-mouse IFN-γ antibody (1:500 dilution in PBS, # 554410, BD Pharmingen), alkaline phosphatase (AKP)-streptavidin conjugate (1:1000 dilution in PBS, # 554065, BD Pharmingen), and staining with 1-Step nitroblue tetrazolium (NBT)–5-bromo-4-chloro-3-indolylphosphate (BCIP) substrate (# B-1911, Sigma). IFN-γ specific spots were counted using an ELISpot reader (ImmunoSpot®), calculated by subtracting the average negative control value, and generated as the net number of spot-forming units (SFUs).

### IFN-γ intracellular cytokine staining

Splenocytes isolated from immunized mice were incubated with peptide HPV16 E7_(49-57)_, D10 (Supplementary Table 1) or I5 (Supplementary Table 2) in the medium with addition of Brefeldin A (GolgiStop, BD) in U-bottom 96 well plates. After 10 h of incubation at 37 °C, cells were stained with anti-mouse CD4-FITC (1:200 dilution, # 553047, BD Pharmingen), CD8-PE (1:100 dilution, # 553033, BD Pharmingen) and IFN-γ-APC (1:200 dilution, # 562018, BD Pharmingen). LIVE/DEAD™ Fixable Yellow Dead Cell Stain Kit (1:1000 dilution, # L34959, Invitrogen) was employed to exclude dead cells. Samples were analyzed using flow cytometer Fortessa (BD, USA).

### Pseudovirion-based neutralization assay (PBNA)

Sera were collected from antigen-immunized mice and were tested for the neutralizing ability against different HPV pseudovirions (PsVs). Briefly, the PBNA employs HPV PsVs carrying a Gaussia-luciferase reporter gene, which is then transduced into Hela T cells in monolayer culture. Initially, 50 µl of sera were titrated in threefold dilution, ranging from 1:50 to 1:12,150, into Dulbecco modified Eagle medium. Then, 50 µl of different types of PsVs mixed with medium (according to the dilution affording 1 million relative luminescence units (RLU) previously determined via transduction assay) were incubated with the serially diluted sera for 15–20 min. Following the incubation period, 50 µl of Hela T cells at a concentration of 2.5 × 10^5^ cells/ml were introduced into the sera-PsVs mixture. The entire setup was then incubated for 48 h in the incubator with 37 °C, 5% CO_2_ and 96% humidity. Afterward, 10 µl of the cell culture supernatant was transferred to another reading plate. Eventually, 100 µl of Gaussia substrate (PJK GmbH, Germany) was added to the supernatant, and then the Gaussia luciferase activity was measured 15 min upon substrate addition. The resulting luminescence signal was measured via Victor Nivo® (PerkinElmer) and analyzed. Serum concentrations (titers) inhibiting 50% of the PsV infection (EC50) were calculated from mean of duplicates. Sera with EC50 values of more than 50 were defined as neutralizing positive samples.

### L2-peptide Enzyme-linked immunosorbent assay (ELISA)

L2-peptide ELISA was utilized to measure anti-L2 antibody levels in the sera of immunized mice, using previously reported methodology^60^. For each assay plate (CorningTM), 50 µl streptavidin (1 mg/ml at 1:300 dilution in MQ water) was added for coating plates overnight at 37 °C. The plate was blocked for one hour at room temperature by blocking milk, which is 1.5% (w/v) milk powder dissolved in 0.3% (v/v) PBS-T (tween 20) solution. Subsequent to three washings using 0.3% PBS-T, each type of HPV L2 peptide (purchased from GenScript Biotech, Netherlands) was added to the plates at a 1:200 or a 1:400 dilution, depending on the optimal dilution, and incubated for one hour at room temperature. After three additional washes with 0.3% PBS-T, serial dilutions of immunized-mice sera were added to the plates, ranging from 1:100 to 1:24300 with threefold titration, and incubation for one hour at 37 °C. Following another washing step, a secondary antibody (goat-anti-mouse IgG conjugated with horseradish peroxidase (JIM-109-035-003, Biozol), at 1:3000 dilution in blocking milk) was added to plates and incubated for one hour at 37 °C. Substrate buffer containing 1 mg/ml 2-2’-azino-di-(3-ethylbenzthiazoline sulfonic acid) (ABTS), 100 mM acetic acid, 50 mM monosodium phosphate, and 0.04% H_2_O_2_, pH 4.2 was prepared and introduced to plates after washing. The resulting emission signal was measured 12–20 min after substrate addition via Multiskan Go (Thermo Fisher Scientific, USA) and analyzed. All titers were calculated by subtracting the background signal, which was determined by adding only PBS instead of sera. L2 peptide-binding antibody titers (IC50) were analyzed according to the serum dilution yielding an average absorbance of 50% of the assay saturation (highest absorbance value) for each peptide.

### Antibody isotype ELISA

Assay plates (CorningTM) were coated with 100 µl of 1 mg/ml streptavidin (Sigma–Aldrich) overnight at 37 °C. After blocked by blocking milk the next day, which is 1.5% (w/v) milk powder dissolved in 0.3% (v/v) PBS-T (tween 20) solution, plates were subsequent to three washings using 0.3% PBS-T. Then 100 µl of diluted N-terminally biotinylated-HPV16 L2 peptide and biotinylated-HPV18 L2 peptide (GenScript)^60^ were added to the plates at a concentration of 2.5 μg/ml and 5 µg/ml, respectively. Following the plate-washing step, pooled serum samples were pipetted into each well at a dilution of 1:50 in 1.5% blocking milk and then titrated in a three-fold serial dilution pattern along the plate. Horse-radish-peroxidase (HRP)-conjugated goat-anti-mouse IgG1 (Southern Biotech 1071-05), IgG2a (Southern Biotech 1081-05), IgG2b (Southern Biotech 1091-05), IgG3 (Southern Biotech 1101-05), IgA (Southern Biotech 1040-05) and IgM (Southern Biotech 1021-05) were used at 1:3000 dilution to determine the isotype of anti-L2 antibodies in sera. The colorimetric reaction was quantified at 405 nm with Multiskan Go (Thermo Fisher Scientific).

### Cell transformation assay

The BRK assay^57^ was performed to evaluate the oncogenic transforming potential of different antigen candidates. Briefly, Baby Rat Kidney (BRK) cells from 9-day-old Wistar rats were seeded at the low confluence and transfected with EJ-ras (2 µg) and the indicated pcDNA plasmids (10 µg). Cells were maintained under selection with G418 (500 µg/ml) for 15–20 days and then colonies were fixed, stained with Giemsa and counted. The plasmid encoding HPV16 E7 is used as a positive control. The empty vector pcDNA is used as a negative control.

### Statistical analysis

Statistical significance was assessed using the nonparametric Mann-Whitney test conducted with GraphPad Prism 8.3.1 (GraphPad Software, USA). A significance threshold of *P* ≤ 0.05 was applied.

### Supplementary information

Supplementary Information

## Data Availability

All the relevant data generated in the study are presented in this manuscript. Additional data are available from the authors upon reasonable request.
