# Supplementary material for: A safe and potentiated multi-type HPV L2-E7 nanoparticle vaccine with combined prophylactic and therapeutic activity
Source: NPJ Vaccines. 2024 Jun 26;9:119. doi: 10.1038/s41541-024-00914-z (PMC11208501; doi:10.1038/s41541-024-00914-z)
Supplement: Supplementary file 1 — Supplementary Information [file 41541_2024_914_MOESM1_ESM.pdf]

Supplementary Materials

Supplementary Tables:

Supplementary Table 1. 'SYFPEITHI'-predicted HLA-DR1-restricted epitopes[1]

Peptide Name	sequence	Score	location of predicted epitope within the antigen components
D1	P D V V N K V E G G G P K T C	36	L2 8mer
D2	S D V I N K V E G G G P M H G	36	L2 8mer
D3	P D I I P K V E G G G P K T C	35	L2 8mer
D4	P D V V P K V E G G G P Q T C	35	L2 8mer
D5	P D V I P K V E G G G P R T C	35	L2 8mer
D6	P D V I P K V E G G G P Q T C	35	L2 8mer
D7	V V L W F S I P G C G P K T C	34	L2 8mer
D8	M G T L G I V C P I C S Q K P	31	E7
D9	V C P I C S Q K P G G P M H G	30	E7
D10	V D K F N I L N V P T L V Y L	30	Trx

[1]Predicted peptide epitopes D1-D10, with scores ≥ 30 were selected for experimental testing.

Supplementary Table 2. OVX313 overlapping peptides[1]

Peptide Name	Sequences
I1	EVGRQNLIRSKEEILKKLKE
I2	RSKEEILKKLKELQEGSKKQ
I3	KLKELQEGSKKQGDADVCGE
I4	SKKQGDADVCGEVAYIQSVV
I5	VCGEVAYIQSVVSDCHVPTA
I6	QSVVSDCHVPTAELRTLLEI
I7	VPTAELRTLLEIRKLFLEIQ
I8	LLEIRKLFLEIQKLKVEGRR
I9	RKLFLEIQKLKVEGRRRRRS

[1]Nine 20-mer peptides, overlapping by 12 residues, spanning the entire sequence of the OVX313 polypeptide.

Supplementary Figures:

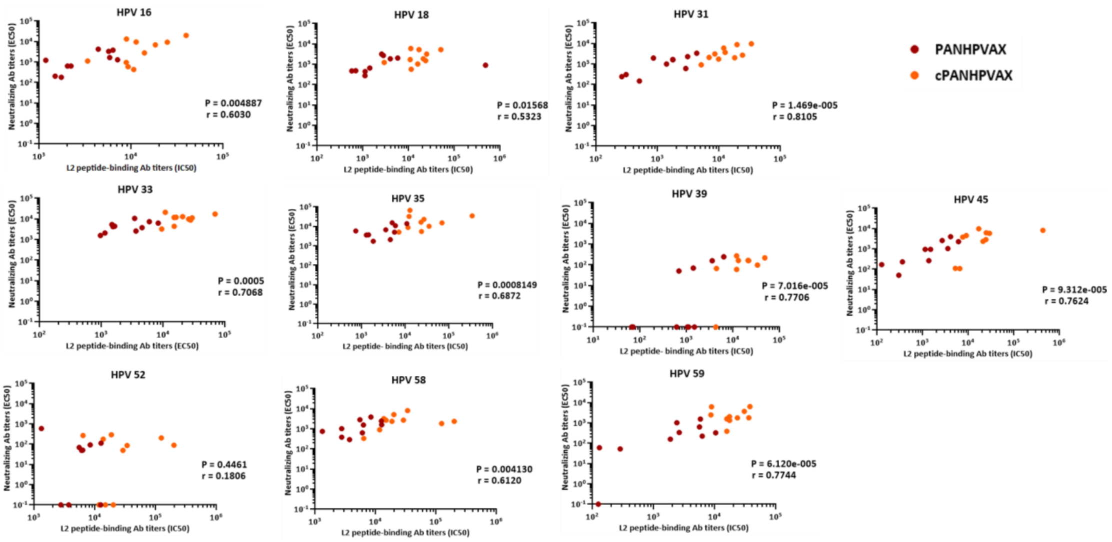

Supplementary Figure 1. Correlation between neutralizing and total L2 peptide-binding antibody titers for 10 different HPV types. Neutralizing antibody titers determined by PBNA (EC50, y-axis) were analyzed for their correlation with the corresponding HPV type specific L2 peptide-binding antibody titers determined by L2-peptide ELISA carried out on individual immune-sera (EC50, x-axis); both measurements were performed on 10 different HPV types, as indicated. Spearman correlation coefficients (r) and *p*-values are indicated in each graph for each HPV type; p-values ≤ 0.05 were considered as statistically significant.

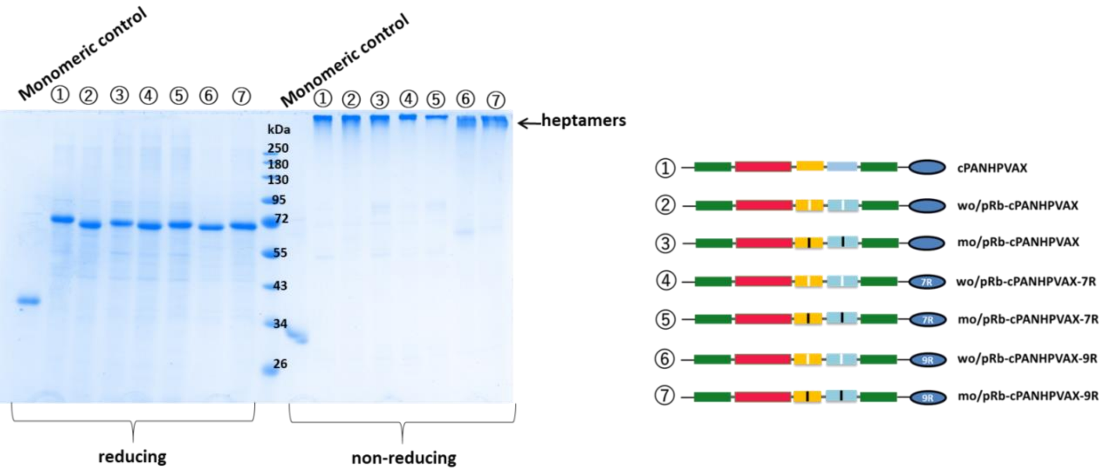

Supplementary Figure 2. SDS-PAGE analysis of the cPANHPVAX antigen and its variants under reducing (*left*) and non-reducing (*right*) conditions. The monomeric control is the previously described PfTrx-$L2_{(20\text{-}38)}$ 8mer-$(OVA_{(257\text{-}264)})_{3X}$ antigen[29]. To be noted is the difference

in electrophoretic mobility between the monomeric (reduced) and the heptameric (non-reduced) form of the antigen, resulting from disulfide bond formation between individual OVX313 units.

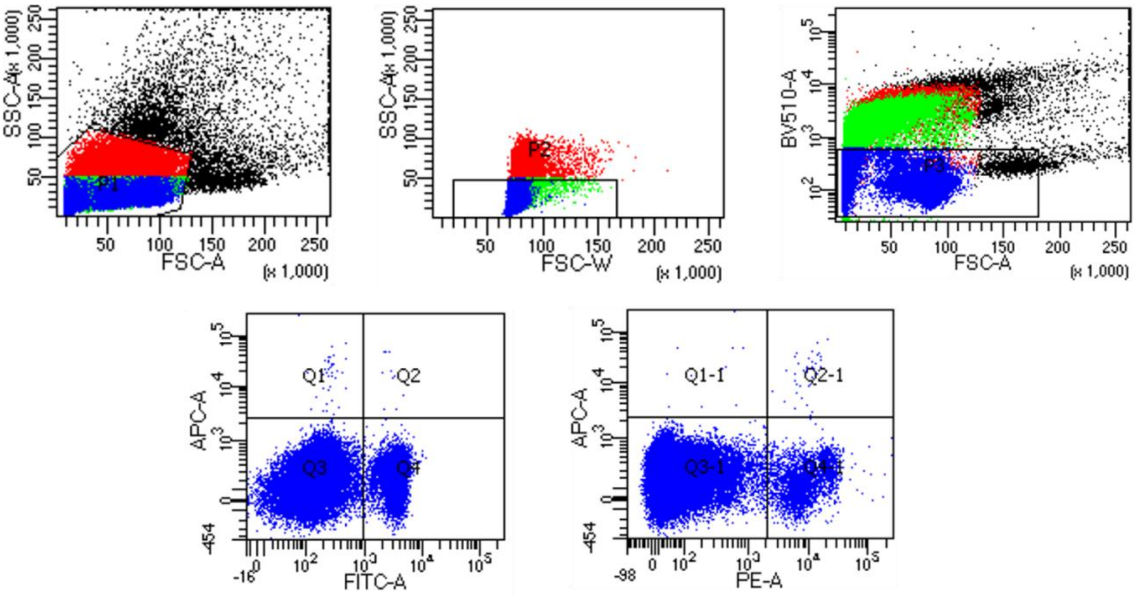

Supplementary Figure 3. Illustration of gating strategy in flow cytometry. The gating example is from peptide D10 stimulated splenocytes derived from a cPANHPVAX immunized A2.DR1 mouse. SSC-A/FSC-A was utilized to gate for the bulk population of cells. SSC-A/FSC-W was employed for the collection of single cells. Subsequently, BV510 (LIVE/DEAD™ Fixable Yellow) negative indicating viable cells were selected. Targeted cell populations included APC+/FITC+ and APC+/PE+.
